# Stereological Estimation of Myocardial Fat and Its Associations with Obesity, Epicardial, and Visceral Adipose Tissue

**DOI:** 10.3390/cells11193160

**Published:** 2022-10-08

**Authors:** Pernille Heimdal Holm, Louise Hindsø, Kristine Boisen Olsen, Jytte Banner

**Affiliations:** 1 Section of Forensic Pathology, Department of Forensic Medicine, University of Copenhagen, 2100 Copenhagen, Denmark; 2 Department of Radiology, University Hospital of Copenhagen, Rigshospitalet, 2100 Copenhagen, Denmark

**Keywords:** myocardial fat, stereology, epicardial adipose tissue, visceral adipose tissue, PMCT

## Abstract

The normal human heart contains epicardial adipose tissue (EAT) and myocardial fat. The associations between obesity, myocardial fat, visceral adipose tissue (VAT), and cardiovascular disease are not fully understood. The objective of this study was to estimate myocardial fat using stereological methods and investigate its relations with obesity, EAT, and VAT. To establish the EAT volume, 115 deceased individuals were included, and postmortem computed tomography was conducted on their eviscerated hearts. Six samples from the left and right ventricles (LV and RV) of the heart were stereologically examined to calculate the percentage of myocardial fat. Kidney and omental fat were weighed at autopsy, and the waist–hip ratio was calculated. Females had a slightly non-significantly (*p* = 0.054) larger proportion of RV fat (13.2% ± 4.4) compared to that in men (11.5% ± 2.7). We found a significant positive correlation between body mass index (BMI) and LV myocardial fat (*p* = 0.033). In the RV, this correlation was only at the borderline of significance (*p* = 0.052). The EAT volume was positively correlated with both RV and LV myocardial fat. We found no association with the waist–hip ratio (WHR) or the omental or kidney fat as measures of VAT. The myocardial fat was normal, most prominent in the RV, and correlated with the EAT and, partly, BMI. We found no association with VAT.

## 1. Introduction

Adipose tissue is present in and around the normal human heart. Epicardial adipose tissue (EAT) is fat tissue around the myocardium without a separation from the fascia. This includes the pericoronary adipose tissue, which surrounds the coronary arteries, extends into the myocardium, and is a type of visceral fat. Visceral adipose tissue (VAT) is fat that is around the organs, unlike subcutaneous and intramuscular fat. This also includes perirenal and omental fat. Myocardial fat comprises bundles of adipocytes that infiltrate the myocardium [1]. There is no consensus in the literature on what to call myocardial fat, and it is referred to as fatty infiltration, adipose infiltration, and intramyocardial fat, among other names. Myocardial fat must not be confused with cardiac steatosis, which is excessive lipid accumulation inside the cardiomyocytes [1,2,3].

Myocardial fat is typically located in the right ventricle (RV) [4], but it is not known whether the amount of myocardial fat in the right ventricle is correlated with myocardial fat in the left ventricle (LV). Increased myocardial fat is seen in different well-established cardiac diseases, and it is hypothesized to be associated with increased arrhythmogenicity [5]. The significance of fatty infiltration in the myocardium is known to occur in arrhythmogenic cardiomyopathy (ACM) and can involve the RV, LV, or both [6]. This association between fat compartments and the risk of different cardiac events is partly known, e.g., in ischemic heart disease and ACM [7]. In some cases of sudden, unexpected death, extensive myocardial fat without an association with a disease unit is even suspected to be the most probable cause of death [8,9]. The significance regarding both the pathophysiology of myocardial fat and the amount of myocardial fat infiltration in these cases is uncertain. Stereological estimations are considered the gold standard for unbiased and objective quantifications in pathology [10]. To our knowledge, no studies have used stereology for the quantification of myocardial fat. 

Classically, obesity is defined by a high body mass index (BMI) and is related to an increase in LV mass [11]. The fat locations and the amount of VAT, however, cannot be assessed with the BMI. Recently, “the obesity paradox” has been recognized, in which a better cardiovascular prognosis is evident in some obese patients, and it has been proposed that obesity phenotypes with different cardiovascular risks exist [11]. The waist–hip ratio (WHR) could be a better estimator for visceral adiposity than the BMI, and VAT could be a better indicator of cardiovascular risk [7]. In addition, EAT has been shown to be an important risk factor for atherosclerosis and cardiovascular disease [12]. However, the associations between obesity (assessed with the BMI), WHR, myocardial fat, EAT, and VAT are not fully understood, and their associations with the risk of heart disease have not been established. 

In this study, we had the unique opportunity to combine whole-body CT scans, CT of eviscerated hearts, autopsy findings, and stereological techniques to assess the relations between BMI and different types of visceral adipose tissue.

The aim of this study was to quantify myocardial fat using stereology and to investigate its correlations with BMI, EAT, and VAT. 

## 2. Materials and Methods

### 2.1. Study Design and Protocol

**Figure 1:**
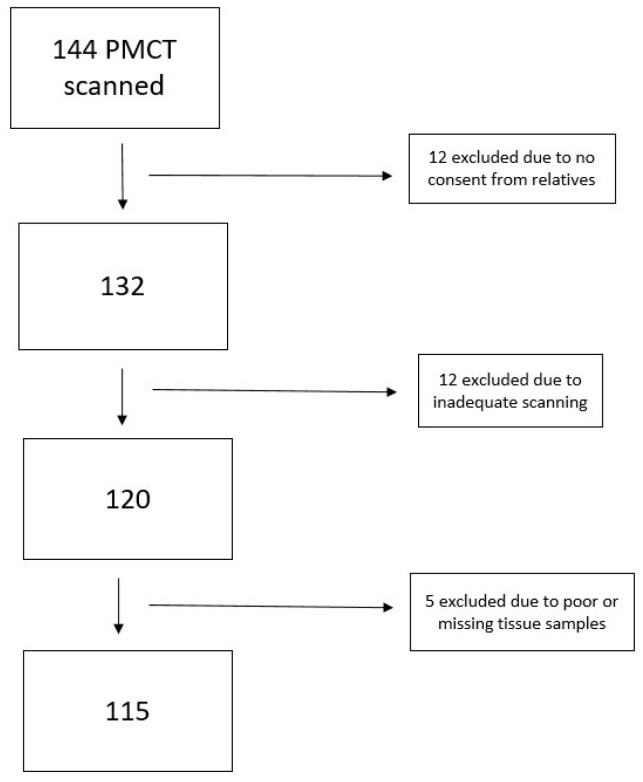
Flowchart of the exclusion process. PMCT = Postmortem Computed Tomography.

This study was a part of the SURVIVE study, a Danish national autopsy study from 2013 to 2015 [13]. The SURVIVE study included 500 deceased individuals who had or were suspected to have severe mental illness, the latter being an internal control group [13]. In this sub-study, we only included cases from the Forensic Institute of Copenhagen with a postmortem computed tomography (PMCT) scan of the eviscerated heart. The exclusion process is illustrated in Figure 1. In total, 115 individuals were included. Informed consent was obtained from all relatives of the subjects involved in the study.

All cases were autopsied according to a standardized and accredited autopsy protocol that followed international guidelines, and the autopsies were extended for sampling regarding both tissue and data. Information regarding the included individuals was collected from police reports. The SURVIVE protocol thoroughly described the registration of physical parameters, organ-specific dissection, and sampling. The height, waist- and hip circumference was measured in the supine position [14] (REF). The data regarding the heart weight, total weight of omental fat, and total weight of kidney fat were registered. The hearts were dissected according to international standards. Histological samples from the hearts were collected from a mid-ventricular slice in the anterior, lateral, and posterior region, of the LV and RV [13]. The samples were embedded in paraffin, sectioned with a thickness of 3 µm, and stained with hematoxylin–eosin (HE). The samples were blinded and whole-slide-scanned with a Hamamatsu scanner at a resolution of x20.

The occurrence of metabolic syndrome was defined with postmortem factors, as described in the article by Christensen et al. [14]. 

### 2.2. Volume Estimation of EAT

PMCT scans were performed by using a standardized scan protocol on a 64-slice Definition Dual-Source CT scanner from Siemens. The total volume of EAT was obtained using the semi-automatic Mimics^®^ software (Materialize, Leuven, Belgium), as described by Hindsø et al. [15].

### 2.3. Stereological Methods

**Figure 2:**
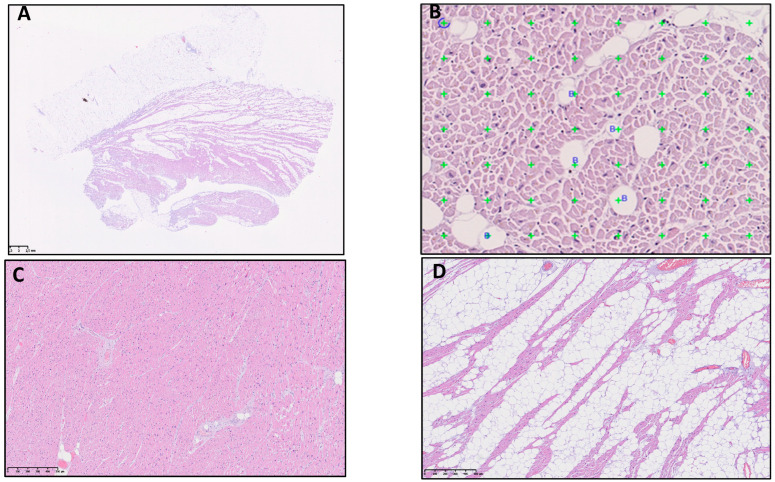
(**A**) Superimage of a whole-slide-scanned image at a resolution of x20. The epicardial adipose tissue (EAT) was excluded in the selection of the region for sampling. (**B**) Example of a sample ROI from the right ventricle (RV) with a 1/56 grid. (**C**) Left-ventricle (LV) sample with small areas of myocardial fat. (**D**) RV sample with extensive myocardial fat.

To determine the myocardial fat, gold-standard stereological principles were applied [10,16]. The stereology was performed by using the Visiopharm software (Visiopharm A/S, Hoersholm, Denmark). A simple point-counting stereology with random regions of interest (ROIs) was used. For the LV samples, we used a 35% random sampling with a 1/56 grid; in the RV, we used 25% sampling of the tissue section with a 1/12 grid. We manually excluded the EAT from the superimage before sampling an ROI. An example is shown in Figure 2A. The orientation of the slides was always with the epicardium, midlayer, and endocardium, so the EAT was clearly visible and could be excluded. The method was tested and optimized to ensure that it was precise considering the big differences between myocardial fat in the right and left ventricles. The sampling percentages of ROIs and the grid ratios needed to be different for the right and left ventricles. A mean of at least 200 counting points of the tissue of interest were used, as suggested by Gundersen et al. [16].

As illustrated in Figure 2, the blue circular point was counted if touching myocardium. Thegreen points were counted when touching an adipocyte. Adipocytes were determined by the presence of a membrane and nucleus. Pericoronary adipose tissue was included as myocardial fat because it was not always possible to distinguish it in the counting frame. The EAT was carefully excluded from the counting. Due to the samples being postmortem, some degree of decomposition was visible. Gaps in the tissue were not counted. 

### 2.4. Statistical Analysis and Ethics

The data were analyzed by using IBM SPSS Statistics for Windows, Version 27.0 Armonk, NY, USA: IBM Corp. To analyze correlations between continuous data, we used the Pearson correlation coefficient. The Mann–Whitney U test was used to analyze differences between groups. A *p*-value < 0.05 was deemed significant. 

The study was approved by the National Committee on Health Research Ethics, Denmark (registration number: 1305373). 

## 3. Results

The final cohort consisted of 57% males and 43% females. The characteristics of the study group are summarized in Table 1. 

The most frequent manners of death were natural and accidental (Table 2). Accidents were dominated by poisonings with prescription medications and/or drugs. Cardiovascular disease was the cause of death in 27% of the population (*n* = 31), with the primary etiology being ischemic heart disease. Only one individual had myocarditis, and no cardiomyopathies were found. No significant differences in myocardial fat in the RV or LV were found between the group with a cardiovascular cause of death and the group with other causes of death (LV: *U* = 1342, *p* = 0.80 and RV: *U* = 1116, *p* = 0.24).

**Figure 3:**
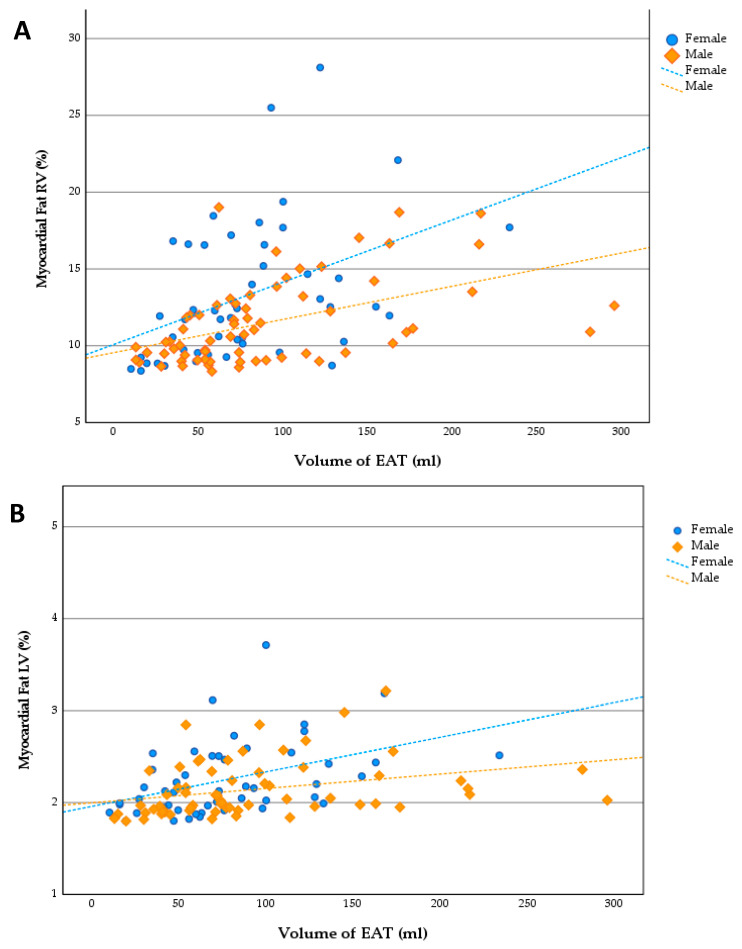

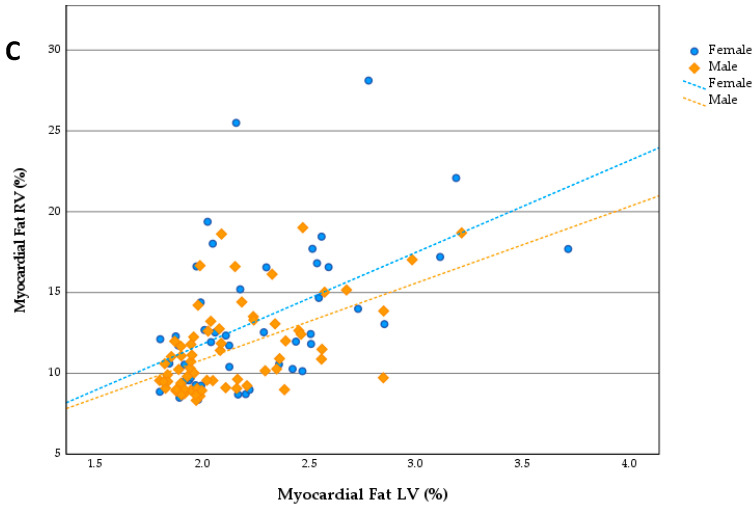
Scatterplots: (**A**) volume of EAT and RV myocardial fat; (**B**) volume of EAT and LV myocardial fat; (**C**) correlation between LV and RV myocardial fat; the combined Pearson correlation coefficient was significant (*r* = 0.54, *p* < 0.01).

The mean of the RV myocardial fat was 12.2% (±3.6), and that for the LV was 2.2% (±0.4). The highest percentage of myocardial fat was in the RV in women (13.2% ± 4.4). Men had a percentage of RV myocardial fat of 11.5% (±2.7). The difference between the sexes was only borderline significant for the RV (*p* = 0.12), and for the LV (*p* = 0.54), the results were not significant. In addition, when analyzing the correlations of age with LV and RV myocardial fat and EAT, no significant differences were found. A significant positive correlation was found between RV and LV myocardial fat (*r* =0.54, *p* < 0.01) (Figure 3C).

A small but significant positive correlation between the BMI and LV myocardial fat (*r* = 0.2, *p* = 0.03) was found; the variance explained was only 4.0%. The correlation of the RV with the BMI was borderline significant (*r* = 0.18, *p* = 0.05). A comparison of the total volume of the EAT and the myocardial fat in both ventricles yielded a moderate significant positive correlation; the variance explained was 10.5% and 14.5% for the LV and RV, respectively (Figure 3A,B, Table 3). The correlations among myocardial fat, EAT, and measures of the VAT (waist–hip ratio, omental fat, and kidney fat) were analyzed, and only the EAT yielded significant correlations with the VAT (Table 3).

Lastly, we examined differences in myocardial fat in the LV and RV and the EAT across groups of individuals with schizophrenia, those taking antipsychotic medicine, or those with metabolic syndrome with a non-parametric Mann–Whitney U test, and we found no significant differences. 

## 4. Discussion

This study stereologically examined myocardial fat in the ventricles and its correlations with obesity, EAT, and VAT. The amounts of myocardial fat in the RV and LV were correlated, and both were associated with EAT. LV myocardial fat and EAT were correlated with the BMI, but RV myocardial fat was not. Only EAT was correlated with all types of VAT.

### 4.1. Myocardial Fat, Sex, and Age

The stereological measurements showed a mean of 12.2% RV myocardial fat and 2.2% LV myocardial fat; the RV and LV myocardial fat levels were correlated. The differences between the sexes and RV myocardial fat were borderline significant, which could have been more evident if our sample size had been larger. Miles et al. [4] used semi-automated image analysis methods to assess myocardial fat. They similarly found a predicted percentage of RV myocardial fat of 12.3% and of LV myocardial fat of 4.7%, and their values of RV and LV myocardial fat were also correlated with each other [4]. They, like others, did not find a difference between males and females [4,17,18]. Tansey et al. found a significantly larger amount of fat in the RV of females who were aged ≥40 years. In younger age groups, they did not find a difference [19].

Older studies have reported a significant increase in myocardial fat with age [17,18,20,21], and it has been suggested that myocardial fat accumulates as part of the normal aging process [19]. In our study, we could not reproduce this difference in age groups. This is in accordance with a few other studies, which did not find an increase in fat with age [4,22].

### 4.2. Myocardial Fat and BMI

In this study, we, like others [4,17] detected a significant correlation between BMI and LV myocardial fat, but not RV myocardial fat. Two studies using CT scans on living subjects did not show a difference between RV myocardial fat and BMI [23,24]; nor did a study that used endomyocardial biopsies [18]. On the contrary, one study reported a significant larger amount of myocardial fat in the RV with increased obesity, but not in the LV [25].

Da Silva et al. examined samples from the LV and found that 20% had myocardial fat [26]. The amount was not assessed. In our study, all individuals had some amount of myocardial fat in the LV, since with stereology, it is possible to detect even very small amounts. We also included pericoronary fat as myocardial fat, though these were separate in their study. They combined myocardial fat, EAT, and pericoronary fat and found correlations with alcoholism, smoking, atherosclerotic disease, acute myocardial infarct, and cardiac hypertrophy. In our study, we did not include these parameters, which could have affected the results. However, Da Silva et al. used a combined value of EAT and myocardial fat, and the EAT might have been an important contributor to the results [26].

### 4.3. Myocardial Fat, Epicardial Adipose Tissue, and Visceral Adipose Tissue

To our knowledge, no autopsy study has studied the relations between several visceral fat storages. EAT and myocardial fat are closely anatomically connected, and as expected, we found a significant correlation with EAT in both the RV and the LV. Surprisingly, only EAT was correlated with all parameters of VAT, but myocardial fat in the LV or RV was not.

Different studies using imaging have looked at myocardial fat and/or cardiac steatosis and their associations with EAT and VAT in the living. One study compared myocardial fat content determined through MR with the thickness of the EAT and waist circumference and showed a significant correlation between both [27], and a study using H-MRS found that cardiac steatoses were related to BMI, visceral fat, and total fat, but found no differences in terms of sex [28]. A CT study on LV intramyocardial fat content showed that it was independently associated with EAT volume [29]. Graner et al. found that intramyocardial fat and EAT were associated with VAT [30]. It is unclear if it is possible to differentiate between myocardial fat and cardiac steatosis with these methods. In this study, we only estimated the myocardial fat and did not include cardiac steatosis. Cardiac steatosis cannot be examined with normal paraffin-embedded tissue sections because the lipids are washed away by fat-soluble agents in the clearing process. Future research should include cardiac steatosis considering the strong associations reported in MR studies and a possible “lipotoxic cardiomyopathy” [7].

EAT was not correlated with age or sex. It was, however, correlated with all types of VAT included in this study. This is surprising, since the myocardial fat in the RV and LV was not correlated with any type of VAT. It is well known that EAT is related to obesity and VAT [31], but, perhaps, myocardial fat is regulated by parameters other than just obesity. Other factors could contribute to the accumulation of myocardial fat, which is not directly related to obesity or VAT. Studies have shown differences in myocardial fat in immunosuppressed patients and patients with HIV [32,33]. When examining differences across groups with schizophrenia, those taking antipsychotic medicine, and/or those with metabolic syndrome, no significant differences were found. However, only a small percentage of the study population had schizophrenia (17%) or metabolic syndrome (14%), and the information regarding diagnosis was not always certain. In this study, we focused on comparing visceral fat depots, but further investigation into mental illness and/or medication and its relation to cardiac fat could, perhaps, yield interesting results.

### 4.4. Strength and Limitations

One of the strengths of this study is the relatively large cohort compared to previous studies. To the best of our knowledge, this is the first study to measure myocardial fat by using gold-standard stereology on cardiac tissue in humans. The strength of stereology is the possibility of estimating ratios from small tissue samples both precisely and reproducibly. The limitation of the stereological method used is the randomization of the counting regions in which myocardial fat cannot be distinguished between the layers (endocardial, midlayer, and epicardial).

The SURVIVE protocol, with its standardized information and thorough sampling of fat tissues, was an essential part of this study. However, the cohort is very selective, and in general, autopsy subjects are a very specific population not necessarily representing the background population. In this study, we chose not to distinguish among severe mental illness, other (aside from cardiac) comorbidities, and the use of medication, though all factors could be important for the development of visceral and/or myocardial fat. However, when comparing cardiovascular causes of death—which are suspected to involve myocardial fat—no significant differences were found in comparison with the other causes of death.

Height, weight, and hip and waist circumferences were measured postmortem in the supine position. This does not always compare to the antemortem measurements. The waist size could be too large due to the development of gases during decomposition; however, one exclusion criterion was moderate to severe decomposition. 

### 4.5. Perspectives

This study has shown how myocardial fat is not correlated with sex, age, or visceral adipose tissue, and only LV myocardial fat showed a correlation with BMI. While LV myocardial fat seems to be associated with obesity, RV myocardial fat does not seem to be. The myocardial fat in both ventricles, however, was associated with EAT, and EAT was correlated with all types of VAT. Perhaps other factors—heart disease, mental illness, or medications—are more important for the accumulation of myocardial fat, which needs further exploration. In our setting, the possibility of combining imaging and autopsy studies could yield interesting results in the exploration of the interconnections among EAT, myocardial fat, and cardiac steatosis.

## Figures and Tables

**Table 1:** Characteristics of the Study Population.

General Characteristics	Total (n = 115)
Men	66 (57%)
Age (years)	53.4 (21–93)
BMI (kg/m^2^)	25.4 ± 5.6
Heart weight (g)	417 (94)
Waist-Hip Ratio	1.0 ± 0.1
Omental fat (g)	233 (300)
Kidney Fat (g)	419 (399)
Schizophrenia	20 (17%)
Antipsychotics	46 (40%)
Metabolic Syndrome	16 (14%)

Values represent means ± SD or (range), median (interquartile range), or n (%). BMI = Body Mass Index.

**Table 2:** Manner and Cause of Death.

Manner of Death	Total (n = 115)
Natural	53 (46.1)
Accident	37 (32.2)
Suicide	16 (13.9)
Undetermined	9 (7.8)
**Causes of Death**	
Poisoning	39 (33.9)
Cardiovascular	31 (27.0)
Pulmonary	2 (1.7)
Infectious	2 (1.7)
Gastrointestinal	5 (4.3)
Endocrine	5 (4.3)
Trauma	18 (15.7)
Chronic Abuse	4 (3.5)
Central Nervous System	2 (1.7)
Neoplasia	2 (1.7)
Unknown	5 (4.3)

Causes of death is grouped based on ICD-10 codes. Values represent n (%).

**Table 3:** Correlations with Myocardial fat, Epicardial Adipose Tissue, and Visceral Adipose Tissue.

Measurements of Fat	LV Myocardial Fat		RV Myocardial Fat		Epicardial Adipose Tissue	
BMI (kg/m^2^)	0.20	**0.03**	0.18	0.05	0.26	**<0.01**
Waist-Hip ratio	0.16	0.08	0.13	0.17	0.29	**<0.01**
Omental fat (g)	0.03	0.72	0.06	0.50	0.41	**<0.01**
Kidney Fat (g)	0.12	0.20	0.05	0.60	0.53	**<0.01**
EAT (ml)	0.32	**<0.01**	0.38	**<0.01**	-	-

Values represents Pearson correlations coefficient and corresponding *p*-values. Significant values are in bold. LV = Left Ventricle, RV = Right Ventricle, BMI = Body Mass Index.

## Data Availability

The data supporting the findings are available from the Department of Forensic Medicine, University of Copenhagen. Access to data is restricted under the license for the study. The data are not publicly available.

## References

[1] Selthofer-Relatić K., Bošnjak I. (2015). Myocardial fat as a part of cardiac visceral adipose tissue: Physiological and pathophysiological view. J. Endocrinol. Investig..

[2] Harmancey R., Wilson C.R., Taegtmeyer H. (2008). Adaptation and Maladaptation of the Heart in Obesity. Hypertension.

[3] Lu Z., Jiang Z., Tang J., Lin C., Zhang H. (2022). Functions and origins of cardiac fat. FEBS J..

[4] Miles C., Westaby J., Ster I.C., Asimaki A., Boardman P., Joshi A., Papadakis M., Sharma S., Behr E.R., Sheppard M.N. (2020). Morphometric characterization of collagen and fat in normal ventricular myocardium. Cardiovasc. Pathol..

[5] Anumonwo J.M.B., Herron T. (2018). Fatty Infiltration of the Myocardium and Arrhythmogenesis: Potential Cellular and Molecular Mechanisms. Front. Physiol..

[6] Neto J.E., Tonet J., Frank R., Fontaine G. (2019). Arrhythmogenic Right Ventricular Cardiomyopathy/Dysplasia (ARVC/D)-What We Have Learned after 40 Years of the Diagnosis of This Clinical Entity. Arq. Bras. Cardiol..

[7] Ferrara D., Montecucco F., Dallegri F., Carbone F. (2019). Impact of different ectopic fat depots on cardiovascular and metabolic diseases. J. Cell. Physiol..

[8] Tuzzolo A., Febres-Aldana C.A., Poppiti R. (2020). Severe Myocardial Steatosis. Am. J. Forensic Med. Pathol..

[9] Kanchan T., Acharya J., Ram P., Khadilkar U., Rana T. (2016). Fat infiltration of left ventricle— A rare cause of sudden cardiac death. Medico-Legal J..

[10] Mühlfeld C., Nyengaard J.R., Mayhew T.M. (2010). A review of state-of-the-art stereology for better quantitative 3D morphology in cardiac research. Cardiovasc. Pathol..

[11] Vecchié A., Dallegri F., Carbone F., Bonaventura A., Liberale L., Portincasa P., Frühbeck G., Montecucco F. (2018). Obesity phenotypes and their paradoxical association with cardiovascular diseases. Eur. J. Intern. Med..

[12] Konwerski M., Gąsecka A., Opolski G., Grabowski M., Mazurek T. (2022). Role of Epicardial Adipose Tissue in Cardiovascular Diseases: A Review. Biology.

[13] Banner J., Høyer C.B., Christensen M.R., Gheorghe A., Bugge A., Ottesen G.L., Boel L.W.T., Thomsen J.L., Kruckow L., Jacobsen C. (2018). SURVIVE: Let the dead help the living—an autopsy-based cohort study for mapping risk markers of death among those with severe mental illnesses. Scand. J. Forensic Sci..

[14] Christensen M.R., Bugge A., Malik M.E., Thomsen J.L., Lynnerup N., Rungby J., Banner J. (2018). Establishing post mortem criteria for the metabolic syndrome: An autopsy based cross-sectional study. Diabetol. Metab. Syndr..

[15] Hindsø L., Jakobsen L.S., Jacobsen C., Lynnerup N., Banner J. (2017). Epicardial adipose tissue volume estimation by postmortem computed tomography of eviscerated hearts. Forensic Sci. Med. Pathol..

[16] Gundersen H.J.G., Bendtsen T.F., Korbo L., Marcussen N., Møller A., Nielsen K., Nyengaard J.R., Pakkenberg B., Sørensen F.B., Vesterby A. (1988). Some new, simple and efficient stereological methods and their use in pathological research and diagnosis. APMIS.

[17] De La Grandmaison G.L., Le Bihan C., Durigon M. (2001). Assessment of right ventricular lipomatosis by histomorphometry in control adult autopsy cases. Int. J. Legal. Med..

[18] Dembinski A.S., Dobson J.R., Wilson J.E., Radio S.J., Miles R.R., Sears T.D., McManus B.M. (1994). Frequency, Extent, and Distribution of Endomyocardial Adipose Tissue: Morphometric Analysis of Endomyocardial Biopsy Specimens from 241 Patients. Cardiovasc. Pathol..

[19] Tansey D.K., Aly Z., Sheppard M.N. (2005). Fat in the right ventricle of the normal heart. Histopathology.

[20] Fontaine G., Fontaliran F., Zenati O., E Guzman C., Rigoulet J., Berthier J.L., Frank R. (1999). Fat in the heart. A feature unique to the human species? Observational reflections on an unsolved problem. Acta Cardiol..

[21] Burke A.P., Farb A., Tashko G., Virmani R. (1998). Arrhythmogenic Right Ventricular Cardiomyopathy and Fatty Replacement of the Right Ventricular Myocardium: Are They Different Diseases?. Circulation.

[22] Selthofer-Relatić K., Belovari T., Bijelić N., Kibel A., Rajc J. (2018). Presence of Intramyocardial Fat Tissue in the Right Atrium and Right Ventricle – Postmortem Human Analysis. Acta Clin. Croat..

[23] Kirsch J., Johansen C.K., Araoz P.A., Brady P.A., Williamson E.E., Glockner J.F. (2010). Prevalence of Fat Deposition Within the Right Ventricular Myocardium in Asymptomatic Young Patients Without Ventricular Arrhythmias. J. Thorac. Imaging.

[24] Jacobi A.H., Gohari A., Zalta B., Stein M.W., Haramati L.B. (2007). Ventricular Myocardial Fat. J. Thorac. Imaging.

[25] Sons H.U., Hoffmann V. (1986). Epicardial fat cell size, fat distribution and fat infiltration of the right and left ventricle of the heart. Anat. Anzeiger..

[26] Da Silva R.M.S., De Mello R.J.V. (2017). Fat deposition in the left ventricle: Descriptive and observacional study in autopsy. Lipids Health Dis..

[27] Malavazos A.E., Di Leo G., Secchi F., Lupo E.N., Dogliotti G., Coman C., Morricone L., Corsi M.M., Sardanelli F., Iacobellis G. (2010). Relation of Echocardiographic Epicardial Fat Thickness and Myocardial Fat. Am. J. Cardiol..

[28] Banerjee R., Rial B., Holloway C.J., Lewandowski A., Robson M.D., Osuchukwu C., Schneider J.E., Leeson P., Rider O.J., Neubauer S. (2015). Evidence of a Direct Effect of Myocardial Steatosis on LV Hypertrophy and Diastolic Dysfunction in Adult and Adolescent Obesity. JACC Cardiovasc. Imaging.

[29] Ng A.C.T., Strudwick M., van der Geest R.J., Ng A.C.C., Gillinder L., Goo S.Y., Cowin G., Delgado V., Wang W.Y.S., Bax J.J. (2018). Impact of Epicardial Adipose Tissue, Left Ventricular Myocardial Fat Content, and Interstitial Fibrosis on Myocardial Contractile Function. Circ. Cardiovasc. Imaging.

[30] Graner M., Sirén R., Nyman K., Lundbom J., Hakkarainen A., Pentikäinen M.O., Lauerma K., Lundbom N., Adiels M., Nieminen M.S. (2013). Cardiac Steatosis Associates With Visceral Obesity in Nondiabetic Obese Men. J. Clin. Endocrinol. Metab..

[31] Rabkin S.W. (2014). The Relationship Between Epicardial Fat and Indices of Obesity and the Metabolic Syndrome: A Systematic Review and Meta-Analysis. Metab. Syndr. Relat. Disord..

[32] Kozman D., Abramowsky C.R., Poulik J., Dereje P., Bondarenko L., Vickery J., Dzul S., Hanan A., Shehata B.M. (2020). Pediatric Right Ventricular Cardiac Steatosis following Immunosuppressive Treatment. Fetal Pediatr. Pathol..

[33] Buggey J., Longenecker C.T. (2017). Heart fat in HIV. Curr. Opin. HIV AIDS.

